# Characteristics and Treatment Outcomes in Advanced-Stage Non-Small Cell Lung Cancer Patients with a *KRAS* G12C Mutation: A Real-World Study

**DOI:** 10.3390/jcm11144098

**Published:** 2022-07-15

**Authors:** Oliver Illini, Hannah Fabikan, Maximilian Johannes Hochmair, Christoph Weinlinger, Dagmar Krenbek, Luka Brcic, Ulrike Setinek, Angelika Terbuch, Gudrun Absenger, Selma Konjić, Arschang Valipour

**Affiliations:** 1 Department of Respiratory and Critical Care Medicine, Klinik Floridsdorf, Vienna Healthcare Group, 1210 Vienna, Austria; maximilian.hochmair@gesundheitsverbund.at (M.J.H.); arschang.valipour@gesundheitsverbund.at (A.V.); 2 Karl Landsteiner Institute for Lung Research and Pulmonary Oncology, Klinik Floridsdorf, Vienna Healthcare Group, 1210 Vienna, Austria; hannah.fabikan@extern.gesundheitsverbund.at (H.F.); christoph.weinlinger@extern.gesundheitsverbund.at (C.W.); 3 Department of Pathology and Bacteriology, Klinik Floridsdorf, 1210 Vienna, Austria; dagmar.krenbek@gesundheitsverbund.at; 4 Diagnostic and Research Institute of Pathology, Medical University of Graz, 8036 Graz, Austria; luka.brcic@medunigraz.at; 5 Institute for Pathology and Microbiology, Klinik Ottakring, Vienna Healthcare Group, 1160 Vienna, Austria; ulrike.setinek@gesundheitsverbund.at; 6 Division of Oncology, Department of Internal Medicine, Medical University of Graz, 8036 Graz, Austria; angelika.terbuch@medunigraz.at (A.T.); gudrun.absenger@medunigraz.at (G.A.); selma.konjic@stud.medunigraz.at (S.K.)

**Keywords:** NSCLC, KRAS G12C mutation, KRAS, lung cancer, real-world data

## Abstract

About 15% of patients with non-small cell lung cancer (NSCLC) harbor the Kirsten rat sarcoma homolog G12C mutation (*KRAS*^G12C^). Selective *KRAS*^G12C^ inhibitors offer new treatment opportunities, but little is known about the prevalence, characteristics, and outcomes of standard-of-care treatment (SOC) in this population. We retrospectively assessed the clinicopathological features of patients with *KRAS*^G12C^-mutated advanced NSCLC and responses to SOC at two high-volume centers in Austria. Out of 2495 NSCLC patients tested, we identified 174 patients with advanced-stage disease carrying a *KRAS*^G12C^ mutation. Most patients were ≥65 years old (55%), heavy smokers (55%), and presented with comorbidities. The most frequent co-alteration was *TP53* (18%). PD-L1 expression was high (TPS ≥ 50%) in 31%, very high (TPS ≥ 90%) in 11%, and negative in 31% of patients. A total of 138 patients (79%) received oncologic systemic treatment. The most common first-line therapy (1 L) was anti-PD-1/PD-L1 plus platinum-based chemotherapy. Median overall survival measured from 1 L treatment was 15.3 months (95% CI, 8.6–21.9), 9.4 (95% CI, 5.3–13.5) from 2 L treatment, and 8.4 (95% CI, 1.7–15.1) from 3 L treatment. The time-to-next-treatment was 8.4 (95% CI, 5.2–11.6) from 1 L and 6.1 (95% CI, 2.7–9.7) months from 2 L to 3 L. These poor outcomes underscore the need for the implementation of new treatment options and for specific molecular testing.

## 1. Introduction

Lung cancer is the leading cause of cancer deaths worldwide [1]. Despite significant advances in the treatment landscape over the last decade, non-small lung cancer (NSCLC), which accounts for 84% of all lung cancer cases, remains a fatal disease for most patients, especially those diagnosed in the advanced stages [2,3].

The rat sarcoma (RAS) oncogene, which exists in three isoforms, is a common oncogenic driver of tumorigenesis in various cancer types. The Kirsten rat sarcoma viral proto-oncogene (*KRAS*) is the most frequently mutated isoform (86%) and almost exclusively present in lung cancer [4]. *KRAS* alterations have been observed in about one quarter of NSCLC patients and occur primarily at codon twelve, where the most prevalent change is a glycine-to-cysteine substitution resulting in the *KRAS*^G12C^ gene mutation [5,6,7,8]. Under physiological circumstances, the membrane-bound guanosine triphosphatase (GTPase) *KRAS* switches from its active GTP-bound state to an inactive GDP-bound state, thereby regulating various signaling pathways (MAPK, PI3K, RalGEF) that are fundamental for cell processes [9]. Point mutations such as G12C lead to a constitutive active state of *KRAS* and drive the uncontrolled cell growth that characterizes cancer. The prevalence of *KRAS*^G12C^ mutations in non-squamous NSCLC has been described as approximately 9–15% [8,10,11].

As compared to other NSCLC alterations, patients with *KRAS*-mutated locally advanced or metastatic NSCLC have had a worse prognosis with shorter overall survival (OS) and progression-free survival (PFS) [10]. The current first-line (1 L) standard-of-care (SOC) therapy for *KRAS*^G12C^-mutant NSCLC is a platinum-based chemotherapy usually combined with immune checkpoint inhibitors (ICIs), depending on the programmed death-ligand 1 (PD-L1) expression status. For patients who progressed on 1 L treatment, chemotherapy with docetaxel, optional in combination with an angiogenic inhibitor, is commonly used as a 2 L therapy. Clinical data revealed a poor median PFS of 2.5–4.0 months for the second or subsequent therapy lines in this patient population [11], indicating an urgent need for new therapeutic agents targeting *KRAS*^G12C^. Nevertheless, *KRAS* has been a difficult target for the development of specific small-molecule inhibitors due to the lack of deep-binding pockets and the need to distinguish between wild-type and mutant *KRAS* [12]. Recently, a breakthrough has been achieved with several selective *KRAS*^G12C^-inhibitors, including sotorasib and adagrasib, showing promising results in early clinical trials [13]. Therefore, sotorasib achieved accelerated approval by the U.S. Food and Drug Administration (FDA) in 2021 [14] and more recently by the European Medicines Agency (EMA) in a second-line setting [15]. With these new drugs becoming widely available for clinical use in the near future, the subpopulation of patients with advanced NSCLC harboring a *KRAS*^G12C^ mutation requires increased attention and understanding to optimize treatment patterns and outcomes.

To this point, data on the characteristics of *KRAS*^G12C^-mutant NSCLC patients have been scarce. The aim of this retrospective cohort study was to describe prevalence, patient characteristics, genetic profiles, prognostic factors, and outcomes of this patient population in a real-world setting.

## 2. Methods

### 2.1. Study Design

This was a retrospective, real-world cohort analysis of patients with advanced (locally advanced or metastatic) *KRAS*^G12C^-mutated NSCLC who were treated at two high-volume referral centers in Austria (the Department of Respiratory and Critical Care Medicine, Clinic Floridsdorf, Vienna, and the Department of Oncology, Medical University of Graz, Graz). The objectives were to describe the demographics, clinical and tumor characteristics, including the genetic tumor profile, their treatment history (surgery, radiotherapy, systemic treatments), as well as treatment responses.

Overall survival (OS) was defined as the time from the start of a given line of treatment until death from any cause; patients without evidence of death were censored with the date of the last follow-up. Additionally, OS was calculated from diagnosis of advanced-stage disease. Time-to-next-treatment (TTNT) was defined as the time between the start of treatment and the start of the subsequent treatment line. Patients without subsequent lines of treatment were censored at the date of last contact or death. Only patients with known treatment start- and end-dates were included for analysis of TTNT.

### 2.2. Study Population and Treatments

The medical records of all consecutive patients with documented advanced *KRAS*^G12C^-mutated NSCLC treated from 1 January 2017 to 31 October 2021 were analyzed. According to institutional practice, NSCLC patients were tested for *KRAS* mutations independently of tumor stage. Between January 2017 and December 2020, obligatory testing for all non-squamous NSCLC was performed. Testing in squamous-cell NSCLC was performed if requested by the treating physicians based on an individual decision. Since January 2021, it was obligatory for all NSCLC patients to be tested for KRAS mutations.

Patients were included if they were ≥18 years old at the initial NSCLC diagnosis, had a histologically confirmed *KRAS*^G12C^ mutation, and underwent treatment and followed-up during the study period. Patients who were initially diagnosed with localized NSCLC were included if they had progressed to an advanced stage until end of the study period.

### 2.3. Data Collection

Predefined clinical characteristics and treatment data were retrospectively extracted from medical records, anonymized by the treating physicians, and transferred for statistical analysis. Collected data included the patients’ characteristics at diagnosis (age, ethnicity, sex, body mass index, smoking history, Charlson comorbidity index score (CCI) [16], Eastern Cooperative Oncology Group (ECOG) performance status, survival status, and tumor characteristics (date of diagnosis, stage, histology, additional mutations found, PD-L1 status and method of testing, location of metastases, method used for detection of *KRAS*^G12C^ mutation)). The type of previously received treatments (surgery, radiotherapy, and systemic therapy), doses, treatment dates, and lines of treatments were gathered, as well as the primary reason for treatment discontinuation. Data collection and data quality control were conducted in accordance with the local institutional standard operating procedures.

### 2.4. Efficacy Assessment

According to institutional practice, a computed tomography (CT) scan of the chest and abdomen was performed every six to twelve weeks to evaluate the tumor response and progression. At the initial diagnosis, additional 15-FDG-PET and/or sonography of cervical lymph nodes and the abdomen were performed as appropriate. Intracranial disease status was usually assessed by brain magnetic resonance imaging (MRI), according to institutional standards.

Real-world efficacy endpoints analyzed were the best tumor response according to RECIST v1.1, assessed by the treating physicians [17], objective response rate (ORR), disease control rate (DCR), TTNT, and OS.

### 2.5. Ethics Approval and Informed Consent

The study protocol was approved by the ethics committee of the city of Vienna, Austria (EK-21-195-VK). Patient consent was waived in agreement with the Institutional Review Board due to the retrospective nature of the study and anonymized data processing. This study was conducted in accordance with the principles of good clinical practice and following the Declaration of Helsinki [18].

### 2.6. Statistical Analysis

Patient demographics as well as clinical, tumor (including genetic mutation profile), and treatment characteristics were summarized descriptively, while categorical data were expressed as frequencies and proportions with 95% two-sided confidence intervals (CI).

Median OS and TTNT were calculated with the Kaplan–Meier method and derived related 95% CI. For survival analyses, the index date was determined by the date of advanced NSCLC diagnosis or the start date of the line of treatment or type of treatment, depending on the analysis. The confidence intervals for proportions such as ORR and DCR were calculated using the exact Clopper–Pearson method.

A Cox proportional hazards model was used to identify factors of possible prognostic influence on overall survival measured from the date of advanced NSCLC diagnosis. The 95% CI for the regression coefficients were based on Wald statistics. All presented *p*-values are two-sided with a level of significance of 5%.

All the statistical analysis was conducted using SPSS v.27.0 (IBM SPSS Statistics, SPSS Inc., Chicago, IL, USA), and the tables and figures were created by using SPSS v.27.0 (IBM SPSS Statistics), Microsoft Excel 2019, and RStudio v.1.4.1106.

## 3. Results

### 3.1. Demographics and Clinical Characteristics

**Figure 1:**
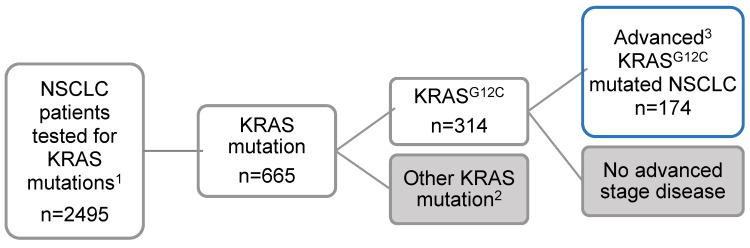
The study population. ^1^ Patients were tested between January 2017 and October 2021. ^2^ Other KRAS mutations are shown in Table 1. ^3^ Patients with NSCLC in locally advanced or metastatic tumor stage.

Overall, 2495 NSCLC patients (independent from tumor stage) were tested for *KRAS* mutations between January 2017 and October 2021 (Figure 1). Next-generation sequencing (NGS)-based genomic profiling from tumor tissue (Thermofisher Oncomine™ Focus assay, Ion AmpliSeq™ Colon, and lung cancer panel or Thermofisher Oncomine™ Sophia DDM analytical platform/IonReporter™ 5.10.5.0) was the method of detection in 97% of the cases. Of all tested patients, 314 (13%) carried a *KRAS*^G12C^ mutation (for prevalence of other *KRAS* mutations, see Table 1). A total of 174 *KRAS*^G12C^-mutated patients had advanced disease and were included for further analysis.

The median age at diagnosis of advanced disease was 66 years (range, 41–87), and approximately half of the patients were ≥65 years old (55%) or male (53%) (Table 2). Most patients were former smokers (55%) or current smokers (38%); 55% were heavy smokers (≥30 pack-years). By proportion, 30% of the patients had ECOG performance status scores of 0, 46% had ECOGs of 1, and 10% had ECOGs ≥ 2. Based on the CCI, 71% of the patients had scores of seven to ten points, and 14% had scores over ten, indicating a high proportion of patients with comorbidities. A history of a previous malignant disease in the last three years was found in 16 patients (9%).

### 3.2. Tumor Characteristics

At initial diagnosis, most of the patients had stage IV (75%) or III (19%) disease (Table 3). Only 5% had early-stage disease at diagnosis and developed disease recurrence later. In patients with advanced-stage disease, the primary sites of metastasis were the bones (29%), brain (27%), lungs (24%), and pleura (21%), followed by the adrenal glands (15%) and liver (11%). In patients with brain metastases (n = 49), most patients (63%) presented with neurologic symptoms. The most common histology found in our study population was adenocarcinoma (90%), followed by 12 cases (7%) with NSCLC-not-otherwise-specified (NOS), two (1%) with neuroendocrine histology, and one with squamous cell carcinoma. High PD-L1 expression (TPS ≥ 50%) was found in 55 patients (31%), and 20 patients (11%) had very high PD-L1 expression (TPS ≥ 90%). PD-L1 was negative in 54 patients (31%).

As assessed by local NGS, in two-thirds (67%) of the patients, no co-mutations were found. The remaining patients had oncogenic driver alterations; the most frequent co-mutation was *TP53* (18%). Four patients had *MET* alterations (2%), one had an *ALK* fusion (1%) and one, a *BRAF*^G464T^ (Exon 11) mutation (1%). In a subpopulation (n = 91) which was tested for *STK11*, the co-mutation was found in 14% of patients.

### 3.3. Treatment History

Overall, 138 patients (79%) with the *KRAS*^G12C^ mutation received at least one systemic treatment line in a palliative setting. At the time of this analysis, 1 L therapy was still ongoing in 19 of those patients. Thirty-six patients (21%) received no cancer-specific systemic treatment. Fifty-six (32%) patients received at least two lines of palliative treatment; in 13 patients, 2 L therapy was ongoing at the time of analysis. Twenty-five (14%) patients received three lines of palliative systemic treatment. As shown in Table 4, 1 L treatment in a palliative setting comprised anti-PD-1/PD-L1 combined with platinum-based chemotherapy in 56 out of 138 patients (41%), platinum-based monotherapy in 45 patients (33%), anti-PD1/PD-L1 monotherapy in 32 (23%), and targeted therapy in five patients (4%). Among 56 patients who received 2 L treatment, the most commonly administered therapy was anti-PD-1/PD-L1 monotherapy in 26 patients (46%), followed by non-platinum-based chemotherapy (mainly docetaxel) in 15 patients (27%), and targeted therapy in 11 patients (20%). As the 3 L treatment (n = 25), targeted therapy (44%) and non-platinum-based chemotherapy/combination (28%) were the most frequent. Only 4% of the patients in 1 L therapy participated in a clinical trial or an expanded access program, versus 23% in 2 L therapy and 40% in 3 L therapy.

Additional information on treatment history, including radiotherapy and surgery with curative or palliative intent, is provided as Supplementary Material (Table S1).

### 3.4. Treatment Responses

A summary of the treatment outcomes is shown in Table 4. The ORR was 44% (95% CI, 34–53) for 1 L systemic therapy, 38% (95% CI, 24–53) for 2 L, and 26% (95% CI, 10–48) for 3 L. The DCR was 66% (95% CI, 56–75) in 1 L, 67% (95% CI, 61–89) in 2 L, and 52% (95% CI, 31–73) in 3 L setting, respectively. As best response, complete response (CR) was achieved in two patients (1%) in the 1 L and one (2%) in the 2 L. Partial response (PR) was reached by 33% (1 L), 29% (2 L), and 24% (3 L) of patients, while 17% (1 L), 23% (2 L), and 24% (3 L) had stable disease (SD).

At the time of analysis, 62 patients were still alive. Median OS from diagnosis of advanced-stage disease (including patients without subsequent oncologic therapy) was 12.7 months (95% CI, 8.7–16.6). Median OS measured from start of the first- (n = 138), second- (n = 56), and third-line (n = 25) therapy was 15.3 months (95% CI, 8.6–21.9), 9.4 (95% CI, 5.3–13.5), and 8.4 months (95% CI, 1.7–15.1), respectively.

There was no difference in median OS between patients with known brain lesions at diagnosis (median OS 12.0 months (95% CI, 7.3–16.7)) and patients without evidence of intracranial disease (median OS 12.9 (95% CI, 7.3–18.4)).

Among 110 patients (patients who received more than one immunotherapy were counted as one) who received immunotherapy at any time, the median OS from the diagnosis of advanced-stage disease was 24.3 months (95% CI, 13.6–34.9). In the 28 patients who never received an immunotherapy, the median OS reached 6.8 months (95% CI, 2.1–11.5); out of those, only five (8%) had 2 L treatment, and one patient (4%) had 3 L treatment.

Similarly, the median OS was 22.0 months (95% CI, 12.6–31.3) for patients who received immunotherapy ± platin-based chemotherapy in 1 L treatment, versus 12.7 months (95% CI, 7.6–17.8) for those who had platin-based chemo-monotherapy.

The median TTNT from the first- to second-line treatment was 8.4 months (95% CI, 5.2–11.6) and from second- to third-line treatment, 6.1 months (95% CI, 2.7–9.7).

The main reason for treatment discontinuation in all treatment lines was the occurrence of disease progression (52% in 1 L, 78% in 2 L, and 61% in 3 L treatment). In the first-line setting, this was followed by the emergence of adverse events (21%), death or reduced general health (15%), and completion of the therapy regimen (8%).

In the multiple regression analysis (Table 5), poorer ECOG, higher CCI, and more than one site of metastases were significantly associated with shorter OS. The presence of a *TP53* co-mutation showed a possible trend for predicting shorter OS, but without statistical significance (*p* = 0.068). A positive (TPS > 1%) PD-L1 status was associated with prolonged OS.

## 4. Discussion

In this retrospective real-world study, we described the prevalence, clinical and pathological characteristics, as well as treatment patterns and efficacy across various lines of SOC therapy in advanced-stage NSCLC harboring a *KRAS*^G12C^ mutation.

The prevalence of *KRAS*^G12C^ mutations in all tested NSCLC patients during the study period was 13%. This finding was overall comparable with previously published data [8]. A slightly higher prevalence of 16% *KRAS*^G12C^ mutations was recently reported in stage IV non-squamous NSCLC patients from the Netherlands (tested in 2017) [20]. A prevalence of *KRAS*^G12C^ mutations of 9% was reported in a retrospective analysis among NSCLC patients in the United States [20,21]. The variability in the prevalence of alterations between regions may partly be explained by differences in risk factors, tumor subtype, and testing strategy. According to institutional practice, most centers in Austria conduct mandatory NGS testing for genetic alterations (reflex testing) for non-squamous NSCLC. Our centers conduct reflex testing also in patients with early tumor stages and, since 2021, in the squamous histologic subtype. With 90% of the patients having adenocarcinoma histology, our data confirmed that *KRAS* mutations were uncommon in other lung cancer subtypes but could even be found in neuroendocrine and squamous cell tumors in rare cases. The fact that until 2021 only selected squamous cell carcinomas were tested for genetic alterations should be considered when interpreting this observation. In addition, ethnic differences in the occurrence of *KRAS* mutations were observed, with Asian populations being less affected than Caucasian populations (5–15% versus 25–50%, respectively [22]); in our population, no patient was Asian.

*KRAS*-mutant NSCLC represents a genetically heterogeneous subgroup with a high frequency of co-occurring mutations in associated pathways, which should be considered when evaluating the treatment outcome [23]. At least at the time of diagnosis, *KRAS* mutations were described to be mutually exclusive with other alterations in NSCLC patients such as *EGFR* and *BRAF* mutations, as well as *ALK* and *ROS* rearrangements [24]. However, rare co-occurrence with *EGFR* (1.2%) and *BRAF* (1.2%) has been found, and co-occurring mutations with *TP53*, *PTEN*, and *STK11* have been described previously [21,23,25,26]. Concurrent mutations could have contributed to the diverse treatment outcomes observed in NSCLC patients harboring *KRAS* mutations. To note, recent data show that patients with *KRAS* and an additional *STK11* mutation have a significantly worse clinical outcome [26], and that the presence of co-mutations such as *STK11* or *KEAP1* might have an impact on treatment efficacy as well [13]. It has been shown that patients with various *KRAS* subtypes have comparable clinical features and treatment outcomes [27]. In our patient pool, the most common co-mutation found by local NGS testing was *TP53* (18%) [20]. However, four cases of *MET* mutations (2%) and one case of an *ALK* (1%) and *BRAF*^G464T^ (1%) mutation were reported. In a subgroup of patients in our study (patients diagnosed at the Medical University of Graz, n = 91), *STK11* was found in 14%.

We described that NSCLC patients harboring *KRAS*^G12C^ were predominantly current or former smokers (55% were heavy smokers) and 55% were above 65 years old; this is in line with previously reported data, as *KRAS*^G12C^ mutations were reported to be associated with age, disease stage, and smoking status [21,25,28]. Additionally, we noted that 40% of the patients had an ECOG ≥1, 45% of them were overweight, and most had a number of comorbidities. These challenges in treating this population may have possibly added to their overall poor prognosis. Previous data have shown that patients with *KRAS*^G12C^ mutations have a lower frequency of lung metastasis than *EGFR*-positive patients do (38% vs. 67%) and a high prevalence of brain metastases (28% at diagnosis and 40% during follow-up) [27,29]. We found that 28% of the patients had pulmonal and 24% had intracranial metastases at diagnosis of advanced-stage *KRAS*^G12C^-mutated NSCLC. One-third of the patients (33%) already had metastases in more than one organ at first diagnosis.

In our study, the most common systemic 1 L palliative treatment regime was anti-PD-1/PD-L1 combined with platinum-based chemotherapy. With an average of 15.3 months, the overall median OS from 1 L treatment was limited, and patients who received only chemotherapy showed poor median OS of 12.7 months. However, the median OS was 22.0 months for patients who received immunotherapy ± platin-based chemotherapy in 1 L treatment, and with a median OS of 24.3 months, patients who received immunotherapy at least once seem to have benefited from this treatment. In particular, the later observation must be interpreted with caution, as only a small portion of patients (8%) who had never received immunotherapy received any subsequent therapy line at all, indicating a poor outcome for this subgroup, independent of therapy regime. However, the outcome of our *KRAS*^G12C^ patients treated with immunotherapy seems fairly good and comparable with the results of metastatic NSCLC patients in the KEYNOTE-189 trial (median OS 22.0 months in the chemo-immunotherapy group) [30].

As expected, the ORR declined from first- to second- and third-line therapies and remained poor in our patients. Likewise, the number of new metastases increased in the later lines of therapy. The TTNT, used as a real-world surrogate marker for PFS, was 8.4 months from the first- to second-line treatment and 6.1 months from second- to third-line treatment. A previously published real-world study found a PFS of 4.7 months in patients with *KRAS*^G12^-mutated NSCLC after 1 L chemotherapy [31]. The difference could be explained by the inherent methodic difference (PFS versus TTNT) but may also be attributed to the common use of immunotherapy in the 1 L treatment in our population.

In multivariate analysis, a reduced performance status (ECOG ≥ 1), multimorbidity (CCI ≥ 7), and more than one site of metastases were independent negative prognostic factors for survival in our patients. A nonsignificant trend for worse survival was also observed for patients with a *TP53* or *STK11* co-mutation. Particularly for *STK11,* the small sample-size and the proportion of patients who were not tested for *STK11* (48%) might have attributed to the results. On the contrary, PD-L1 positivity (TPS > 1%) was identified as a positive prognostic marker for OS. Interestingly, a recent exploratory study suggested that the *KRAS*^G12C^-mutation could be associated with a prolonged response to 1 L immunotherapy in PD-L1-overexpressing NSCLC [32]. PD-L1 is a relevant biomarker in NSCLC, but its role in *KRAS*^G12C^-mutated cancers is not yet clear. Whereas a higher proportion of PD-L1-positive tumors was previously reported in *KRAS*^G12D, G12V^ or *KRAS*^G13C^ subtypes, PD-L1-negative tumors were predominantly observed with *KRAS*^G12A^ and *KRAS*^G12C^ mutations [33]; therefore, immunotherapies might be effective for selected *KRAS*-mutated tumors only [13]. In addition, the sensitivity to chemotherapy might differ across subtypes of a KRAS mutation [34]. In our study, *KRAS*^G12C^ mutations did not appear to be associated with an excessively high PD-L1 expression. We found that 31% of *KRAS*^G12C^-mutated NSCLC showed PD-L1 expression ≥50% (TPS), and 65% had TPS ≥ 1%, which appeared to be in line with previously published data on *KRAS*^G12C^ NSCLC (34% with TPS) and unselected NSCLC patients (22–30%, 52–63%, respectively) [35]. Moreover, smoking behavior is associated with a high tumor mutational burden (TMB) and could predict a better response; in a recent large retrospective analysis, the authors described that *KRAS*^G12C^-mutated lesions were significantly associated with a high TMB status [21]. However, most phase III clinical trials evaluating all those with NSCLC treated with immunotherapy did not stratify by *KRAS* status, and only post hoc analyses provided information regarding their efficacy. With respect to pembrolizumab, an exploratory analysis from the KEYNOTE-042 trial showed an improved median OS and PFS in *KRAS*-mutated versus *KRAS* wild-type tumors [36]. In the KEYNOTE-189 trial, platinum-based chemotherapy alone or combined with pembrolizumab as the 1 L treatment demonstrated improved efficacy, regardless of *KRAS* status [37]. However, Frost et al. investigated the efficacy of 1 L pembrolizumab in patients with *KRAS*-mutated lung adenocarcinoma with high PD-L1 expression (TPS ≥ 50%) and showed a significantly higher ORR and longer PFS in patients harboring *KRAS*^G12C^/*TP53* co-mutations, as compared to patients with *KRAS*^non-G12C^/*TP53* mutations [28]; these results enhanced the importance of assessing *KRAS* subtypes and *TP53* mutations before pembrolizumab therapy. Other data have shown a comparable efficacy of immunotherapy in NSCLC patients with *KRAS*^G12C^-mutations or presenting with other genetic alterations [33,38,39]. PD-L1 expression could, therefore, be more relevant for predicting immunotherapy efficacy in *KRAS*-mutant NSCLC than in other NSCLC tumors [33].

In our study, 36 patients (20%) had not received any systemic treatment based on poor clinical status or patient decision. Only about half of our patients received 2 L therapy, with anti-PD-1/PDL-1 monotherapy being the most common. Despite the inherent limitations of this retrospective study, that observation indicates poor prognosis and a rapid deterioration of health status in *KRAS*^G12C^ patients and could encourage attempts to implant selective KRAS inhibitors in early-line settings in the future.

The outcomes underscore the need for new treatment options such as RAS GTPase family inhibitors. The novel *KRAS*^G12C^ inhibitors sotorasib and adagrasib have shown promising efficacy results in early clinical trials. Sotorasib, a small molecule that traps *KRAS*^G12C^ in the inactive GDP-bound state, was investigated in the phase I/II study CodeBreak 100 (NCT03600883) in patients with advanced solid tumors harboring a *KRAS*^G12C^ mutation. In the subgroup of NSCLC patients, an ORR was observed in 37% of the patients, while the PFS reached 6.8 months, and the OS was 12.5 months [40]. Sotorasib was therefore granted accelerated approval by the U.S. FDA for adult patients with *KRAS*^G12C^-mutant locally advanced or metastatic NSCLC who had received at least one prior systemic therapy [14]. Eventually, sotorasib was approved as the first targeted therapy for patients with *KRAS*^G12C^-mutated advanced NSCLC who had progressed after at least one prior line of systemic therapy by the EMA in January 2022 [15]. A global phase III trial, CodeBreak 200 (NCT04303780), comparing sotorasib with docetaxel in patients with *KRAS*^G12C^-mutated NSCLC, is ongoing [9]. Adagrasib, another potent, covalent KRAS inhibitor that selectively binds *KRAS*^G12C^, demonstrated encouraging clinical activity in the phase I/II KRYSTAL-1 trial (NCT03785249); in pretreated NSCLC patients with *KRAS*^G12C^ mutations, the ORR was 45% [9]. Adagrasib received a breakthrough designation by the FDA for treating metastatic lung cancer patients harboring *KRAS*^G12C^ who have progressed after at least one prior systemic therapy [13].

This retrospective analysis carried several inevitable limitations, such as selection bias, reporting bias, and information bias. Moreover, given the small sample size and no internal comparator, only descriptive efficacy outcomes have been presented. In addition, clinicopathological characteristics and treatment outcomes only have been described for patients with *KRAS*^G12C^ mutation and not in comparison with other major subtypes like *KRAS*^G12D^ and *KRAS*^G12V^.

Our results provided a deeper understanding on the eligibility of patients and necessity for treatment with upcoming selective *KRAS*^G12C^ inhibitors. The poor outcome of our patient population emphasized the urgent need for targeted treatment options. The availability of these new therapies reinforces the importance of molecular testing and early detection of *KRAS*^G12C^ in clinical practice. A recently published nationwide retrospective cohort study in the Netherlands reported an increase in the national testing rate for *KRAS* mutations in non-squamous NSCLC, from 70% in 2013 to 82% in 2017; the development of new technologies such as NGS certainly favored this promising tendency [20]. In Austria, reflex testing for *KRAS* mutations is strongly recommended by national guidelines in non-squamous NSCLC but could be performed for patients with other histologic subtypes in individual cases [41]. To provide our patients with the best opportunity to receive precision medicine, the mandatory and broad use of NGS testing should be ensured, and furthermore, we recommend testing in all NSCLC subtypes.

## Figures and Tables

**Table 1:** Occurrence of different KRAS mutations in 2495 NSCLC patients.

KRAS Mutation, n (%) ^1^	All Patients (N = 665)
G12C	314 (47.2)
G12V	103 (15.5)
G12D	86 (12.9)
G12A	40 (6.0)
G13D	23 (3.5)
G13C	23 (3.5)
Q61H	16 (2.4)
G12S	15 (2.3)
G12F	9 (1.4)
G12R	6 (0.9)
G13V	4 (0.6)
Q61L	3 (0.5)
Q61R	2 (0.3)
L19F	2 (0.3)
A146T	2 (0.3)
E63K	2 (0.3)
G12H	1 (0.2)
G12I	1 (0.2)
G12Y	1 (0.2)
G13Dup	1 (0.2)
G13E	1 (0.2)
G13Y	1 (0.2)
G13delinsAGG	1 (0.2)
Q22K	1 (0.2)
P34R	1 (0.2)
A146P	1 (0.2)
A59E	1 (0.2)
T35P	1 (0.2)
A146V	1 (0.2)
K5N	1 (0.2)
P34R	1 (0.2)

^1^ Including patients in all tumor stages tested between January 2017 and October 2021. Until January 2021, reflex testing for KRAS mutations was performed only for non-squamous NSCLC. The percentage may not be equal to 100 due to rounding.

**Table 2:** Demographics and clinical characteristics.

Demographics and Clinical Characteristics ^1^	All Patients (N = 174)
**Age**, years	
Median (range)	66 (41–87)
Age groups, n (%)	
*<65*	77 (44)
*≥65*	96 (55)
*Unknown*	1 (1)
**Sex**, n (%)	
Male	93 (53)
Female	81 (47)
**Race**, n (%)	
Non-Asian	174 (100)
**Smoking status**, n (%)	
Never smoker	6 (3)
Former smoker	96 (55)
Current smoker	66 (38)
Unknown	6 (3)
**Pack-years**, n (%)	
Never Smoker **(<30 py)**	30 (17)
Heavy smoker **(≥30 py)** ^2^	95 (55)
Unknown	43 (25)
*Range*	3–150
**Performance status (ECOG ^3^)**, n (%)	
0	53 (30)
1	80 (46)
≥2	18 (10)
Unknown	23 (13)
**Body Mass Index (BMI)**, n (%)	
Underweight (<18.5)	9 (5)
Normal range (18.5–24.9)	67 (39)
Overweight (25.0–29.9)	50 (29)
Obese Class I (30.0–34.9)	23 (13)
Obese Class II (35.0–39.9)	5 (3)
Unknown	20 (11)
**Charlson Comorbidity Index Score** ^4^, n (%)	
0	0 (0)
1–3	6 (3)
4–6	20 (11)
7–10	124 (71)
>10	24 (14)
**History of other cancer (last 3 years)**, n (%)	
Yes	16 (9)
No history	116 (67)
Unknown	42 (24)

NA, not applicable; py, pack-years. ^1^ Characteristics at diagnosis of advanced or metastatic disease. The percentage may not equal to 100 due to rounding. ^2^ As defined by the National Lung Screening Trial [19]. ^3^ ECOG (Eastern Cooperative Oncology Group) performance status, with higher numbers indicating increasing impairment in daily life activities at diagnosis of advanced stage disease. ^4^ Calculated with the Charlson comorbidity index [16].

**Table 3:** Tumor characteristics.

Tumor Characteristics ^1^	All patients (N = 174)
**Stage at initial diagnosis**, n (%)	
Stage I	3 (2)
*Stage Ia*	*2 (1)*
*Stage Ib*	*1 (1)*
Stage II	6 (3)
*Stage IIa*	*1 (1)*
*Stage IIb*	*5 (3)*
Stage III	33 (19)
*Stage IIIa*	*7 (4)*
*Stage IIIb*	*17 (10)*
*Stage IIIc*	*9 (5)*
Stage IV	131 (75)
Unknown	1 (1)
**Location of metastasis** ^2^, n (%)	
Bones	51 (29)
Brain	47 (27)
Lungs	42 (24)
Pleura	36 (21)
Adrenal glands	26 (15)
Liver	20 (11)
Other	15 (9)
Unknown	1 (1)
**Sites of metastases** ^2^, n (%)	
None	16 (9)
1	99 (57)
2–3	35 (20)
>3	23 (13)
Unknown	1 (1)
**Brain metastasis**, n (%)	N = 49
Asymptomatic	9 (18)
Symptomatic	31 (63)
Unknown	9 (18)
**Histology subtype**, n (%)	
Adenocarcinoma	157 (90)
NSCLC NOS ^3^	12 (7)
Squamous Cell Carcinoma	1 (1)
Neuroendocrine Tumor	2 (1)
Other	2 (1)
**PD-L1 status (TPS %)**, n (%)	
Negative (<1%)	54 (31)
1–49%	59 (34)
50–89%	35 (20)
≥90%	20 (11)
Unknown	6 (3)
**Other genetic alterations in NGS**, n (%)	
Patients without findings	116 (67)
Patients with findings	57 (33)
*TP53*	*31 (18)*
*ALK fusion*	*1 (1)*
*BRAF G464T Exon 11*	*1 (1)*
*MET* ^3^	*4 (2)*
*Other findings* ^4^	*30 (17)*
Unknown	1 (1)

NGS, next-generation sequencing; NOS, not otherwise specified. ^1^ The percentage may not equal to 100 due to rounding. ^2^ Concerning patients with disease stage IIIb/c and IV. ^3^ Including MET Exon 14 T1010I, MET Exon 14 Deletion, MET NM_001127500. ^4^ Including AR mutation, CTNNB1, DDR2, ERBB4, GNAS, MAP2K1, MYC amplification, SMAD4 and STK11. STK11 was tested only in a subgroup of 91 patients and was found in 14% of those.

**Table 4:** Treatment patterns and responses.

Treatment Patterns and Responses in Palliative Setting ^1^	1st Line N = 138	2nd Line N = 56	3rd Line N = 25
**Type of systemic therapy**, n (%)			
Anti-PD-1/PD-L1 and platinum-based chemotherapy	56 (41)	2 (4)	1 (4)
Platinum-based chemotherapy alone	45 (33)	1 (2)	2 (8)
Anti-PD-1/PD-L1 monotherapy	32 (23)	26 (46)	4 (16)
Targeted therapy ^2^	5 (4)	11 (20)	11 (44)
Non-platinum-based chemotherapy/combination	0 (0)	15 (27)	7 (28)
Other	0 (0)	1 (2)	0 (0)
**Clinical trial or expanded access program**^3^, n (%)			
Yes	6 (4)	13 (23)	10 (40)
**Objective response rate (ORR)**^4^, % (95% CI)	44 (34–53)	38 (24–53)	26 (10–48)
**Disease control rate (DCR)**^5^, % (95% CI)	66 (56–75)	67 (61–89)	52 (31–73)
**Best response**, n (%)			
Complete response (CR)	2 (1)	1 (2)	0 (0)
Partial response (PR)	45 (33)	16 (29)	6 (24)
Stable disease (SD)	24 (17)	13 (23)	6 (24)
Progressive disease (PD)	37 (27)	15 (27)	11 (44)
Not evaluable/Unknown	30 (22)	11 (20)	2 (8)
**New metastasis at start of therapy**, n (%)			
Yes	20 (14)	25 (45)	9 (36)
No	105 (76)	30 (54)	14 (56)
Unknown	13 (9)	1 (2)	2 (8)
**Primary reason for treatment discontinuation**, n (%)	N = 109	N = 40	N = 23
Progressive disease	57 (52)	31 (78)	14 (61)
Adverse event	23 (21)	3 (8)	2 (9)
Death or reduced general health	16 (15)	4 (10)	3 (13)
Completed regimen	9 (8)	1 (3)	0 (0)
Other	2 (2)	0 (0)	1 (4)
Unknown	2 (2)	1 (3)	3 (13)
**Time to next treatment (TTNT)** ^6^	N = 72	N = 36	-
Median, months (95% CI)	8.4 (5.2–11.6)	6.1 (2.7–9.7)	
**Overall survival (OS)** ^7^	N = 133	N = 54	N = 25
Median, months (95% CI)	15.3 (8.6–21.9)	9.4 (5.3–13.5)	8.4 (1.7–15.1)

^1^ The percentage may not equal to 100 due to rounding. ^2^ Including alectinib (2), capmatinib (1), sotorasib (2). ^3^ Patients who participated in a clinical trial or an expanded access program were not included for the calculation of systemic-therapy-specific ORR, OS, and TTNT. ^4^ ORR was defined as complete or partial response assessed by the treating physicians; patients with an unknown response were excluded. ^5^ DCR included complete response, partial response, or stable disease; patients with an unknown response were excluded. ^6^ TTNT was defined as the time between the start of systemic treatment to the first dose of following systemic treatment; only patients with a known start date were analyzed. Patients who did not receive further treatment, e.g., due to death, were excluded. ^7^ OS was calculated from the start of systemic treatment to the date of death, regardless of cause. Patients who were alive or lost to follow-up were censored at the last date known alive. Patients with an unknown start date were excluded.

**Table 5:** Cox multiple regression analysis for overall survival.

Variable	HR	95% CI	*p*-Value
**Sex**			
Female vs. Male	1.469	0.973–2.219	0.067
**Age**			
<65 vs. ≥65 years	0.794	0.48–1.293	0.354
**ECOG** ^1^			<0.001
1	1.744	1.005–3.027	0.048
2	3.694	1.884–7.241	<0.001
3	15.540	5.417–44.579	<0.001
**Heavy smoker** *(≥30 py)* ^2^			
Yes vs. No	0.956	0.633–1.444	0.830
**Charlson Comorbidity Index Score**			0.011
<7 vs. 7–9	5.051	1.673–15.251	0.004
<7 vs. ≥10	5.915	1.824–19.182	0.003
**Sites of metastases**	3.312	2.158–5.082	<0.001
<1 vs. >1			
**Brain metastases**			
No/Unknown vs. Yes	0.894	0.578–1.383	0.614
**TP 53 co-mutation**			
No vs. Yes	1.596	0.966–2.637	0.068
**STK11 co-mutation** ^3^			
No/Unknown vs. Yes	1.813	0.853–3.852	0.122
**PD-L1**			
Negative vs. >1% TPS	0.513	0.330–0.798	0.003

HR, hazard ratio; py, pack-years; PD-L1, programmed death-ligand. ^1^ ECOG (Eastern Cooperative Oncology Group) performance status, with higher numbers indicating increasing impairment in daily life activities at diagnosis of advanced stage disease. ^2^ As defined by the National Lung Screening Trial [19] ^3^ STK11 was tested only in a subgroup of 91 patients. Patients with an STK11 co-mutation were tested against patients negative for STK11 or with unknown STK11 status.

## Data Availability

The data presented in this study are available on request from the corresponding author. The data are not publicly available due to the valid European General Data Protection Regulations.

## Supplementary Materials

The following supporting information can be downloaded at: https://www.mdpi.com/article/10.3390/jcm11144098/s1. Table S1. Treatment history.

## References

[1] Sung H., Ferlay J., Siegel R.L., Laversanne M., Soerjomataram I., Jemal A., Bray F. (2021). Global Cancer Statistics 2020: GLOBOCAN Estimates of Incidence and Mortality Worldwide for 36 Cancers in 185 Countries. CA Cancer J. Clin..

[2] Miller M., Hanna N. (2021). Advances in systemic therapy for non-small cell lung cancer. BMJ.

[3] Herbst R.S., Heymach J.V., Lippman S.M. (2008). Lung cancer. N. Engl. J. Med..

[4] Cox A.D., Fesik S.W., Kimmelman A.C., Luo J., Der C.J. (2014). Drugging the undruggable RAS: Mission possible?. Nat. Rev. Drug Discov..

[5] Tate J.G., Bamford S., Jubb H.C., Sondka Z., Beare D.M., Bindal N., Boutselakis H., Cole C.G., Creatore C., Dawson E. (2019). COSMIC: The Catalogue Of Somatic Mutations In Cancer. Nucleic Acids Res..

[6] Prior I.A., Lewis P.D., Mattos C. (2012). A comprehensive survey of Ras mutations in cancer. Cancer Res..

[7] Duma N., Santana-Davila R., Molina J.R. (2019). Non-Small Cell Lung Cancer: Epidemiology, Screening, Diagnosis, and Treatment. Mayo Clin. Proc..

[8] Biernacka A., Tsongalis P.D., Peterson J.D., de Abreu F.B., Black C.C., Gutmann E.J., Liu X., Tafe L.J., Amos C.I., Tsongalis G.J. (2016). The potential utility of re-mining results of somatic mutation testing: KRAS status in lung adenocarcinoma. Cancer Genet..

[9] Reck M., Carbone D.P., Garassino M., Barlesi F. (2021). Targeting KRAS in non-small-cell lung cancer: Recent progress and new approaches. Ann. Oncol..

[10] Goulding R.E., Chenoweth M., Carter G.C., Boye M.E., Sheffield K.M., John W.J., Leusch M.S., Muehlenbein C.E., Li L., Jen M.-H. (2020). KRAS mutation as a prognostic factor and predictive factor in advanced/metastatic non-small cell lung cancer: A systematic literature review and meta-analysis. Cancer Treat. Res. Commun..

[11] Hayashi H., Okamoto I., Taguri M., Morita S., Nakagawa K. (2013). Postprogression survival in patients with advanced non-small-cell lung cancer who receive second-line or third-line chemotherapy. Clin. Lung Cancer.

[12] Salgia R., Pharaon R., Mambetsariev I., Nam A., Sattler M. (2021). The improbable targeted therapy: KRAS as an emerging target in non-small cell lung cancer (NSCLC). Cell Rep. Med..

[13] Cucurull M., Notario L., Sanchez-Cespedes M., Hierro C., Estival A., Carcereny E., Saigí M. (2021). Targeting KRAS in Lung Cancer Beyond KRAS G12C Inhibitors: The Immune Regulatory Role of KRAS and Novel Therapeutic Strategies. Front. Oncol..

[14] (2021). Sotorasib FDA Approval. https://www.fda.gov/drugs/resources-information-approved-drugs/fda-grants-accelerated-approval-sotorasib-kras-g12c-mutated-nsclc.

[15] (2022). Sotorasib EMA Approval. https://www.ema.europa.eu/en/medicines/human/EPAR/lumykras.

[16] Charlson M.E., Pompei P., Ales K.L., MacKenzie C.R. (1987). A new method of classifying prognostic comorbidity in longitudinal studies: Development and validation. J. Chronic Dis..

[17] Eisenhauer E.A., Therasse P., Bogaerts J., Schwartz L.H., Sargent D., Ford R., Dancey J., Arbuck S., Gwyther S., Mooney M. (2009). New response evaluation criteria in solid tumours: Revised RECIST guideline (version 1.1). Eur. J. Cancer.

[18] World Medical Association (2013). World Medical Association Declaration of Helsinki: Ethical principles for medical research involving human subjects. JAMA.

[19] de Koning H.J., Van Der Aalst C.M., De Jong P.A., Scholten E.T., Nackaerts K., Heuvelmans M.A., Lammers J.-W.J., Weenink C., Yousaf-Khan U., Horeweg N. (2020). Reduced Lung-Cancer Mortality with Volume CT Screening in a Randomized Trial. N. Engl. J. Med..

[20] Garcia B.N.C., van Kempen L.C., Kuijpers C., Schuuring E., Willems S.M., van der Wekken A.J. (2022). Prevalence of KRAS p.(G12C) in stage IV NSCLC patients in the Netherlands; a nation-wide retrospective cohort study. Lung Cancer.

[21] Salem M.E., El-Refai S.M., Sha W., Puccini A., Grothey A., George T.J., Hwang J.J., O’Neil B., Barrett A.S., Kadakia K.C. (2022). Landscape of KRAS(G12C), Associated Genomic Alterations, and Interrelation With Immuno-Oncology Biomarkers in KRAS-Mutated Cancers. JCO Precis. Oncol..

[22] Karachaliou N., Mayo C., Costa C., Magrí I., Gimenez-Capitan A., Molina-Vila M.A., Rosell R. (2013). KRAS mutations in lung cancer. Clin. Lung Cancer.

[23] Scheffler M., Ihle M.A., Hein R., Merkelbach-Bruse S., Scheel A.H., Siemanowski J., Brägelmann J., Kron A., Abedpour N., Ueckeroth F. (2019). K-ras Mutation Subtypes in NSCLC and Associated Co-occuring Mutations in Other Oncogenic Pathways. J. Thorac. Oncol..

[24] Jacobs F., Cani M., Malapelle U., Novello S., Napoli V.M., Bironzo P. (2021). Targeting KRAS in NSCLC: Old Failures and New Options for “Non-G12c” Patients. Cancers.

[25] Liu Y., Li H., Zhu J., Zhang Y., Liu X., Li R., Zhang Q., Cheng Y. (2021). The Prevalence and Concurrent Pathogenic Mutations of KRAS (G12C) in Northeast Chinese Non-small-cell Lung Cancer Patients. Cancer Manag. Res..

[26] El Osta B., Behera M., Kim S., Berry L.D., Sica G., Pillai R.N., Owonikoko T.K., Kris M.G., Johnson B.E., Kwiatkowski D.J. (2019). Characteristics and Outcomes of Patients With Metastatic KRAS-Mutant Lung Adenocarcinomas: The Lung Cancer Mutation Consortium Experience. J. Thorac. Oncol..

[27] Cui W., Franchini F., Alexander M., Officer A., Wong H.-L., Ijzerman M., Desai J., Solomon B.J. (2020). Real world outcomes in KRAS G12C mutation positive non-small cell lung cancer. Lung Cancer.

[28] Frost N., Kollmeier J., Vollbrecht C., Grah C., Matthes B., Pultermann D., von Laffert M., Lüders H., Olive E., Raspe M. (2021). KRAS(G12C)/TP53 co-mutations identify long-term responders to first line palliative treatment with pembrolizumab monotherapy in PD-L1 high (≥50%) lung adenocarcinoma. Transl. Lung Cancer Res..

[29] Wu M., Zhang E., Strickland M., Mendoza D., Lipkin L., Lennerz J., Gainor J., Heist R., Digumarthy S. (2021). Clinical and Imaging Features of Non-Small Cell Lung Cancer with G12C KRAS Mutation. Cancers.

[30] Gadgeel S., Rodríguez-Abreu D., Speranza G., Esteban E., Felip E., Dómine M., Hui R., Hochmair M.J., Clingan P., Powell S.F. (2020). Updated Analysis From KEYNOTE-189: Pembrolizumab or Placebo Plus Pemetrexed and Platinum for Previously Untreated Metastatic Nonsquamous Non-Small-Cell Lung Cancer. J. Clin. Oncol..

[31] Lei L., Wang W.-X., Yu Z.-Y., Liang X.-B., Pan W.-W., Chen H.-F., Wang L.-P., Fang Y., Wang M., Xu C.-W. (2020). A Real-World Study in Advanced Non-Small Cell Lung Cancer with KRAS Mutations. Transl. Oncol..

[32] Cefalì M., Epistolio S., Ramelli G., Mangan D., Molinari F., Martin V., Freguia S., Mazzucchelli L., Froesch P., Frattini M. (2022). Correlation of KRAS G12C Mutation and High PD-L1 Expression with Clinical Outcome in NSCLC Patients Treated with Anti-PD1 Immunotherapy. J. Clin. Med..

[33] Jeanson A., Tomasini P., Souquet-Bressand M., Brandone N., Boucekine M., Grangeon M., Chaleat S., Khobta N., Milia J., Mhanna L. (2019). Efficacy of Immune Checkpoint Inhibitors in KRAS-Mutant Non-Small Cell Lung Cancer (NSCLC). J. Thorac. Oncol..

[34] Mellema W.W., Masen-Poos L., Smit E.F., Hendriks L., Aerts J.G., Termeer A., Goosens M.J., Smit H.J., Heuvel M.M.V.D., van der Wekken A.J. (2015). Comparison of clinical outcome after first-line platinum-based chemotherapy in different types of KRAS mutated advanced non-small-cell lung cancer. Lung Cancer.

[35] Dietel M., Savelov N., Salanova R., Micke P., Bigras G., Hida T., Antunez J., Skov B.G., Hutarew G., Sua L. (2019). Real-world prevalence of programmed death ligand 1 expression in locally advanced or metastatic non-small-cell lung cancer: The global, multicenter EXPRESS study. Lung Cancer.

[36] Herbst R.S., Lopes G. (2019). Association of KRAS Mutation Status with Response to Pembrolizumab Monotherapy Given as First-Line Therapy for PD-L1 Positive Advanced Non-Squamous NSCLC in KEYNOTE-042. Ann. Oncol..

[37] Gadgeel S., Rodriguez-Abreu D. (2019). KRAS Mutational Status and Efficacy in KEYNOTE-189: Pembrolizumab Plus Chemotherapy (vs Placebo Plus Chemo as First-Line Therapy for Metastatic Non-Squamous NSCLC. Ann. Oncol..

[38] Sebastian M., Eberhardt W.E., Hoffknecht P., Metzenmacher M., Wehler T., Kokowski K., Alt J., Schütte W., Büttner R., Heukamp L.C. (2021). KRAS G12C-mutated advanced non-small cell lung cancer: A real-world cohort from the German prospective, observational, nation-wide CRISP Registry (AIO-TRK-0315). Lung Cancer.

[39] Spira A.I., Tu H., Aggarwal S., Hsu H., Carrigan G., Wang X., Ngarmchamnanrith G., Chia V., Gray J.E. (2021). A retrospective observational study of the natural history of advanced non-small-cell lung cancer in patients with KRAS p.G12C mutated or wild-type disease. Lung Cancer.

[40] Skoulidis F., Li B.T., Dy G.K., Price T.J., Falchook G.S., Wolf J., Italiano A., Schuler M., Borghaei H., Barlesi F. (2021). Sotorasib for Lung Cancers with KRAS p.G12C Mutation. N. Engl. J. Med..

[41] Pirker R., Prosch H., Popper H., Klepetko W., Dieckmann K., Burghuber O.C., Klikovits T., Hoda M.A., Zöchbauer-Müller S., Filipits M. (2021). Lung Cancer in Austria. J. Thorac. Oncol..

