# Supplementary material for: Characteristics and Treatment Outcomes in Advanced-Stage Non-Small Cell Lung Cancer Patients with a KRAS G12C Mutation: A Real-World Study
Source: J Clin Med. 2022 Jul 15;11(14):4098. doi: 10.3390/jcm11144098 (PMC9324356; doi:10.3390/jcm11144098)
Supplement: Supplementary file 1 [file jcm-11-04098-s001.zip › jcm-1780355-supplementary.pdf]

Supplementary Materials

This Supplementary Materials section has been provided by the authors to give readers additional information about their work.

Oliver Illini et al.

Title: Characteristics and treatment outcomes in advanced stage non-small cell lung cancer patients with KRAS G12C mutation: a real-world study

Supplement Table S1 – Treatment history

**Treatment history**[1]	**All patients** (N=174)
**Prior anticancer therapy**, n (%)	
Neoadjuvant therapy	10 (6)
Adjuvant therapy	8 (5)
Surgery	30 (17)
Segmentectomy or Wedge resection	*4 (13)*
Bilobectomy or Lobectomy	*18 (60)*
Pneumonectomy	*2 (7)*
Removal of metastasis and other	*6 (20)*
**Radiotherapy**, n (%)	
Curative	11 (6)
Palliative	87 (50)
Unknown	4 (2)
No radiation	74 (43)
**Location of radiotherapy**, n (%)	N=100
Brain	38 (38)
Lungs	28 (28)
Lymph nodes	5 (5)
Other	32 (32)
Unknown	1 (1)

[1] Percentage may not equal to 100 because of rounding
